# Patterns of patient worry following major emergency abdominal surgery: a 180-day follow-up study

**DOI:** 10.1007/s00068-026-03287-1

**Published:** 2026-07-29

**Authors:** Malthe Kirk Parner, Lasse Rehné Jensen, Hejdi Gamst-Jensen, Jakob Burcharth, Dunja Kokotovic

**Affiliations:** 1 Emergency Surgery Research Group Copenhagen (EMERGE), Department of Hepatic and Gastrointestinal Diseases, Copenhagen University Hospital - Herlev and Gentofte, Herlev, Denmark; 2 Department of Clinical Medicine, University of Copenhagen, Copenhagen, Denmark; 3 Department of Anesthesia Centre of Head, Neck and Orthopedics, Copenhagen University Hospital - Rigshospitalet, Copenhagen, Denmark; 4 Department of Surgery, Holbaek Hospital, Region Zealand, Holbaek, Denmark; 5 Department of Surgery, Copenhagen University Hospital - Herlev and Gentofte, Herlev, Denmark

**Keywords:** Major emergency abdominal surgery, Degree-of-Worry (DOW), Days Alive and Out of Hospital (DAOH), Postoperative recovery, Psychological distress, Patient-reported outcome measures (PROM)

## Abstract

**Purpose:**

Major emergency abdominal surgery is associated with high postoperative morbidity and mortality. Beyond physical complications, patients experience psychological distress. This study aimed to describe the distribution of patient-reported worry across independent cross-sectional samples and its association with *Days Alive and out of Hospital* (DAOH) over 180 days postoperatively.

**Methods:**

Prospective cohort at Copenhagen University Hospital Herlev (March 30, 2023–March 31, 2024). *Degree of Worry* (DOW) was surveyed at discharge and *postoperative day* (POD) 30, 90, and 180, categorized as physical, mental, and physical-generic domains of worry. DAOH was computed cumulatively and incrementally for 0–30, 30–90, and 90–180 days. Associations between *high DOW* (hDOW) and subsequent DAOH were tested with Mann–Whitney U.

**Results:**

hDOW was reported by 36.1% at discharge, 22.4% at POD 30, 45.0% at POD 90, and 32.0% at POD 180. Across 180 days, the most frequent worries were relapse (21.3%), disease (14.1%), functional level (12.0%) and daily living (9.1%). The proportion of physical worries was significantly higher at POD 30 than at discharge and significantly lower at POD 180; mental worries varied between time points without statistically significant differences. hDOW at POD 30 was associated with fewer cumulative DAOH by POD 90 (*p* = 0.008).

**Conclusion:**

Patient-reported worry following major emergency abdominal surgery demonstrates meaningful variation over time. Physical worries peak during early recovery, whereas mental worry varied across timepoints. hDOW at POD 30 is associated with reduced DAOH by POD 90, highlighting the relevance of monitoring patient-reported worry during early postoperative recovery.

**Supplementary Information:**

The online version contains supplementary material available at 10.1007/s00068-026-03287-1.

## Introduction

Major emergency abdominal surgery indicated for conditions such as perforated hollow viscus, bowel obstruction, mesenteric ischemia and complicated acute abdominal wall disorders (e.g., burst or open abdomen), is associated with high rates of postoperative morbidity and mortality with mortality rates of ~ 20% at 30 days and ~ 34% at 1 year [1–4]. Recovery following major emergency abdominal surgery is often prolonged and complex. In the first postoperative week, patients typically face significant limitations in physical performance, with fatigue and abdominal pain acting as primary barriers to independent mobilization [5]. Post-discharge, nearly 50% of patients require emergency readmission within 180 days, reflecting the high burden of postoperative complications [6]. Long-term outcomes are similarly concerning, with a two-year mortality rate of nearly 20% among patients surviving the first 90 days postoperatively [7]. Even at one year, many patients report persistent pain and functional impairments [8]. Beyond physical consequences, these patients experience a significantly higher prevalence of psychological distress, including depression and anxiety, compared to the general population [9]. Understanding patients’ worries in the early postoperative period is crucial, as these worries potentially has a profound impact on rehabilitation and the overall course of recovery. Early identification and management of these worries may influence long-term outcomes, potentially improving both physical and psychological patterns of recovery.

A tool to quantify degree and subjects of worry is the *Degree of worry* (DOW) -questionnaire. The self-reported DOW captures patients’ subjective perceptions of illness severity and urgency [10]. As of now, DOW has been applied in triaging patients contacting medical helplines, such as Denmark’s 1813 service, and within emergency department settings, where it has shown potential as a supportive tool in clinical assessment and decision-making [10–15]. No studies have examined the subjects- or patterns of worry quantified by DOW following hospital discharge.

This study aims to describe the degree and subjects of worry among patients undergoing major emergency abdominal surgery across predefined postoperative timepoints (discharge, day 30, 90, 180) during the first 180 days postoperatively. Additionally, the study seeks to identify and categorize the specific subjects of worry expressed by patients at different recovery stages and assess the association between *High DOW* (hDOW) and patient outcomes, including *Days Alive and Out of Hospital* (DAOH) [16] and readmission.

## Methods

### Study design and population

This study was designed as a prospective cohort study including patients undergoing major emergency abdominal surgery at Copenhagen University Hospital Herlev. The hospital is a tertiary care center with an acute care function, serving a population of approximately 425,000 individuals [17]. The study period spanned from March 30, 2023, to March 31, 2024. All participants included in the study were categorized within the *Acute High-risk Abdominal Surgery* (AHA) framework [18]. AHA is a treatment model utilized in Denmark for managing patients with severe intra-abdominal surgical conditions that necessitate major emergency abdominal surgery. This care bundle provides a structured approach to the assessment and treatment of patients requiring urgent surgical intervention, ensuring standardized and comprehensive care pathways [18].

### Degree of worry

DOW is a tool developed for medical triage at a medical helpline for acute non-emergent conditions. The DOW-tool was developed to bring the caller’s perception of the severity of their condition into time constrained telephone consultations that are known for closed questions and quick report [12, 13]. The DOW may aid call handlers to understand the patient’s self-evaluated sense of urgency, offering a subjective measure to support clinical assessment [12–14]. DOW is scaled along a continuum from problem solving coping, where individuals seek information and solutions, to emotional coping, marked by distress and fear of consequences [19].

The DOW scale was previously defined on a range from 1 to 10 to mitigate a flooring effect in calls to a medical helpline (where patients are supposed to experience a DOW). For this study the DOW scale was expanded from 0 to 10, as this modification was expected to provide a more detailed and nuanced insight into the patients’ levels of DOW postoperatively. This was in collaboration with the DOW developers.

The original DOW scale was developed in the context of acute medical helpline contacts, where some degree of worry is inherent in the decision to seek urgent advice. In the present postoperative follow-up setting, however, patients may plausibly report no current worry. Therefore, the DOW scale was expanded from 1 to 10 to 0–10 to allow patients to indicate absence of worry and to reduce the risk of a floor effect. This modification was made in collaboration with the DOW developers. For the purpose of this study, hDOW was defined a priori as a score of ≥ 6. This threshold was established in collaboration with the DOW developers and was chosen to represent the upper range of the scale, corresponding to a clinically meaningful level of worry warranting attention in the postoperative setting. As this threshold has not been formally validated for postoperative patients, it should be interpreted as an exploratory cutoff. In addition to the numerical score, all patients were asked to describe up to three main subjects of worry related to the numerical score they expressed. To structure the qualitative subjects of patient-reported worries, an exploratory framework was developed inductively by the research team. Reported subjects of worry were grouped into 14 predefined categories based on thematic similarity and clinical relevance. These categories were subsequently consolidated into three overarching domains. Coding of patient-reported subjects of worry into the 14 predefined categories was performed by one investigator (M.K.P.). In cases of uncertainty, coding decisions were discussed with a senior investigator to ensure agreement on domain placement. The consolidation of categories into three overarching domains was subsequently discussed and agreed upon by all five authors through clinical consensus, ensuring that domain assignments reflected both clinical relevance and collective agreement. Formal inter-rater reliability was not assessed, as the categorization was intended as an exploratory framework rather than a validated classification system. The physical domain was comprised of subjects of worry related to immediate bodily or clinical issues. These were “pain, procedure, test results, relapse, disease, stoma, postoperative symptoms, and death”. The physical-generic domain incorporated subjects of worry related to broader health and functional level. These were “diet/food intake and functional level.“. Lastly the mental domain covered subjects of worry reflecting psychosocial or cognitive stressors including “mental status, the future, daily living, and unknowingness during or after hospitalization and rehabilitation regarding the postoperative course.” Here, “daily living” was included as a mental domain, as patients emphasized the psychological burden of managing everyday functioning under uncertainty.

This tripartite categorization was developed to ensure a holistic understanding of patient worry, with the physical domain covering clinical and somatic aspects, the mental domain addressing emotional and cognitive dimensions, and the physical-generic domain capturing broader lifestyle and functional issues.

### Ethical considerations

The study was approved by the Capital Region of Denmark (protocol numbers P-2020-1166 and R-21038079) and the Danish Data Protection Agency (approval number P-2021-431). The study adhered to the principles outlined in the STROBE checklist for observational studies [20].

### Data collection

Patient-reported outcomes were collected through the DOW-questionnaire administered either verbally at discharge or via telephone at POD 30, POD 90 and POD 180. Some patients were not assessed at discharge and therefore provided their first DOW assessment at POD 30. As not all patients responded at every follow-up, DOW responses at discharge, POD 30, POD 90, and POD 180 represent independent samples at each timepoint and were therefore analyzed as cross-sectional observations rather than within-subject repeated measures. Follow-up data also included all-cause mortality and hospital readmissions, retrieved from patient records. Verbal informed consent was obtained from all participants prior to inclusion, either during the in-person interview or at the beginning of the telephone contact. If a patient was unreachable, a second attempt was made within the following days; if no response was obtained, no further attempts were made until the next scheduled follow-up. Contacting patients by phone after discharge was approved by the Danish Medical Research Ethics Committee (H-21027246).

Patient-data were extracted from the patients’ electronic record and registered using the *Research Electronic Data Capture* (REDCap) platform. The dataset comprised a range of clinical and patient-reported variables, including baseline characteristics (e.g., age, sex, *Body Mass Index* (BMI) and *American Society of Anesthesiologists* (ASA) grade), preoperative and intraoperative data (e.g., medication usage, history of abdominal surgery, and perioperative findings or strategies), and postoperative complications (categorized by organ-specific location). The severity of postoperative complications was classified according to the *Clavien-Dindo* (CD) classification system, which categorizes complications based on the type of intervention required, ranging from minor (Grade I) to life-threatening or fatal (Grade V) [21]. Frailty was assessed using the *Clinical Frailty Scale* (CFS), a validated 9-point scale measuring patients’ baseline vulnerability to adverse health outcomes. CFS scores range from 1 (very fit) to 9 (terminally ill) and were determined at the time of admission based on clinical judgment, chart review, and patient/family interviews when necessary [22].

All data were collected and managed using the REDCap platform, hosted by the Department of Gastrointestinal and Hepatic Diseases at Copenhagen University Hospital – Herlev and Gentofte, Denmark. REDCap is a secure, web-based platform designed to support research data capture [23].

### Outcome measures

The primary outcome of the study was the proportion of patients with hDOW assessed at predefined postoperative timepoints during the first 180 days after discharge. Comparisons were performed between discharge and POD 30, between POD 30 and POD 90, and between POD 30 and POD 180.

Secondary outcomes included the distribution of patient-reported subjects of worry categorized into three domains: (1) physical, (2) mental, and (3) physical-generic. These secondary outcomes were also compared across the same three timepoints to describe domain-specific patterns.

The tertiary outcome examined the association between hDOW and two postoperative recovery outcomes: DAOH and hospital readmission.

Cumulative DAOH was calculated as the total number of days a patient was alive and not hospitalized at POD 30, 90, and 180, according to established methodology [16]. Incremental DAOH intervals were calculated to reflect recovery rate over time rather than cumulative burden alone: DAOH_0-30_ (discharge to POD 30), DAOH_30-90_, and DAOH_90-180_. To facilitate interpretation of postoperative recovery, thresholds were predefined to identify low recovery based on cumulative DAOH at each timepoint. Low recovery was defined as DAOH fewer than 15 days by POD 30, fewer than 60 days by POD 90, and fewer than 120 days by POD 180. These thresholds were chosen to reflect substantially reduced time at home relative to the maximum possible DAOH at each timepoint. For readmission analyses, hDOW assessed at each timepoint was compared with subsequent hospital readmissions. Specifically, hDOW at discharge was compared with readmissions within 30 days, hDOW at POD 30 with readmissions within 90 and 180 days, and hDOW at POD 90 with readmissions within 180 days. This analysis aimed to explore the temporal relationship between patients’ DOW and the probability of hospital readmission.

### Statistical analyses

Descriptive analyses were conducted to summarize the data. Median values with *interquartile ranges* (IQRs) were reported due to non-normal distribution of variables. Because DOW data were not paired across timepoints, comparisons of DOW scores over time were based on independent samples rather than within-subject repeated measures.

Within-subject changes in DAOH across postoperative time intervals were assessed using Friedman’s test, with post hoc pairwise comparisons performed using Wilcoxon signed-rank tests with Bonferroni correction (α = 0.017). Friedman’s test was applied to DAOH because this outcome was derived from longitudinal hospital records and therefore constituted paired observations. Associations between hDOW and subsequent DAOH were analyzed using Mann–Whitney U tests at discharge, POD 30, POD 90, and POD 180. Each hDOW timepoint was matched to the following DAOH interval (e.g., hDOW 30 → DAOH 30–90). Effect sizes were reported as *r = |Z|/√N*. Sensitivity analyses used cumulative DAOH as the outcome. Associations between categorical variables (e.g., hDOW and readmission) were assessed using Pearson’s chi-squared (χ²) test.

A p-value of < 0.05 was considered statistically significant. All analyses were conducted in SPSS (IBM Corp., Armonk, NY, USA).

## Results

**Fig. 1:**
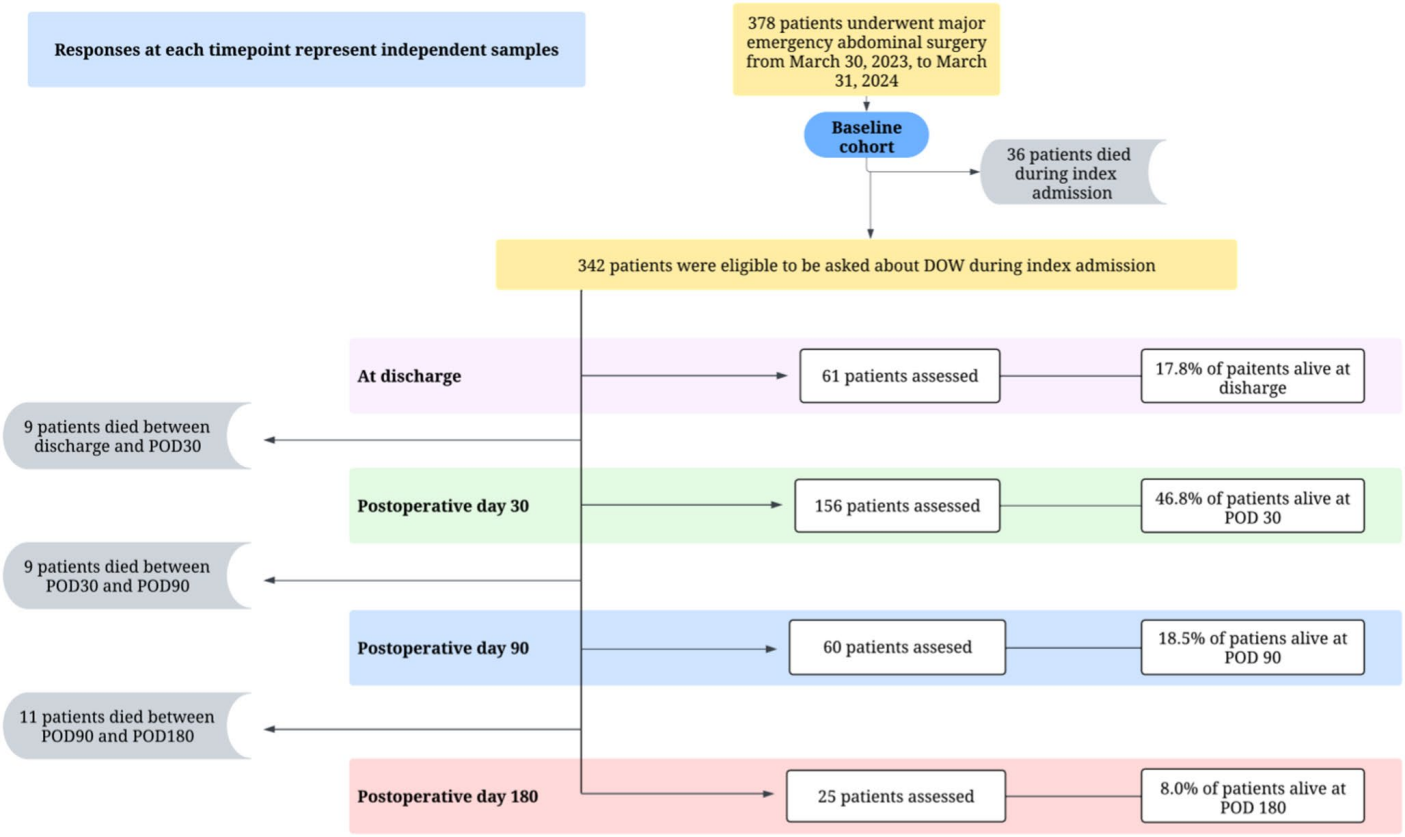
Flowchart of the study population and response rates for DOW assessments. DOW responses at discharge and POD 30, 90, and 180 represent independent cross-sectional samples. Patients responding at each timepoint were not necessarily the same individuals. Response rates are shown as percentages of eligible patients at each timepoint

Flowchart of patient inclusion are presented in Fig. 1.

A total of 378 patients were included in the study with a median age of 72.9 (IQR 58.3–80.7) of which 43.7% were male. The majority of patients (47.9%) had a CFS of 4 categorized as “vulnerable”. A total of 87.3% of patients underwent a primary operation, with 53.2% receiving open surgery, of which 95.7% were midline laparotomies. Half of the patients (50%) were operated for bowel obstruction, followed by 22.0% for perforation, and 19.0% for other indications.

Psychiatric disorders were present in 10.0% of patients. Among these, 61.1% had anxiety and/or depression, 8.3% had schizophrenia, 8.3% had bipolar disorder, and 13.9% were diagnosed with other psychiatric conditions. Further information about the patient cohort, is presented in Table 1.

**Table 1:** Demographics and clinical characteristics

Patient Characteristics	
**Number of patients (N=)**	378
**Sex**,** male (N=/%)**	165 (43.7)
**Age**,** median (interquartile range)**,** years**	72.9 (58.3–80.7)
**Body Mass Index (kg/m2)**,** median (IQRs)**	24.9 (21.5–28.3)
**ASA physical status (N=/%)**	
I-II	210 (55.6)
III	146 (38.6)
IV-V	22 (5.8)
**WHO performance score (N=/%)**	
0–1	299 (79.1)
2–3	68 (18.0)
4–5	3 (0.8)
**Clinical Frailty Scale (N=/%)**	
1–3	47 (12.4)
4–6	307 (81.2)
7–9	22 (5.8)
**Organ specific comorbidities (N=/%)** ^**a**^	
Cerebral	52 (13.8)
Cardiovascular	210 (55.7)
Peripheral vascular	14 (3.7)
Pulmonary	69 (18.3)
Endocrinological	62 (16.4)
Gastrointestinal	42 (11.1)
Renal	19 (5.0)
Rheumatological	41 (10.9)
**Metastasized malignant disease (N=/%)**	39 (10.3)
**Psychiatric disorders** ^**b**^ **(N=/%)**	36 (10.0)
Anxiety and/or depression	22 (61.1)
Schizophrenia	3 (8.3)
Bipolar disorder	3 (8.3)
Other	5 (13.9)
**Lifestyle factors (N=/%)**	
Smoking	85 (23.2)
Alcohol	33 (9.1)
Other	6 (2.0)
**Living arrangements and social status (N=/%)**	
Own home	358 (96.8)
Protected residence	4 (1.1)
Nursing home	8 (2.2)
Domestic help	61 (16.9)
Living alone	150 (41.8)
Married/in relationship	200 (55.6)
**Previous abdominal surgery (N=/%)**	181 (48.0)
Midline incision	64 (35.4)
**Operative characteristics (N=/%)**	
Primary operation	254 (87.3)
Reoperation	37 (12.7)
Laparoscopic	77 (20.5)
Laparoscopic converted to open	99 (26.3)
Open	200 (53.2)
Midline laparotomy^c^	286 (95.7)
**Intraoperative pathology (N=/%)**	
Obstruction	189 (50.0)
Perforation	83 (22.0)
Ischemia	14 (3.7)
Fascial dehiscence	6 (1.6)
Incarcerated/strangulated hernia	28 (7.4)
Other^d^	72 (19.0)
**Postoperative course (N=/%)**	
Postoperative complications (CD ≥ 2)	265 (70.3)
Postoperative ICU stay	66 (17.6)
Length of stay, median (interquartile range), days	6 (4–13)
Reoperation during admission	66 (17.6)
** Mortality** ^**e**^	65 (17.2)

Values are number of patients (%) unless stated otherwise. ASA = American Society of Anaesthesiologists. WHO = World Health Organization

a= Organ specific comorbidities include:

Cerebral: TCI, stroke, Alzheimer/dementia, epilepsy and other

Cardiac: Angina pectoris, previous AMI, Chronic ischemic heart disease, Heart insufficiency, Heart failure, medically treated hypertension, hypercholesterolemia, atrial fibrillation

Peripheral vascular comorbidity: Chronic peripheral ulcers, intermittent claudication

Pulmonary: COPD (chronic obstructive pulmonary disease), Asthma, Pulmonary fibrosis, Other

Endocrinological: Diabetes mellitus type 1. Diabetes mellitus type 2 diet-regulated/medically regulated, Thyroid disease, pituitary/adrenal disease, other

Gastrointestinal: Liver cirrhosis, Chronic hepatitis, Ulcerative colitis, Crohn’s disease, Chronic pancreatitis, irritable colon, other

Renal: Chronic renal failure, Diabetic nephropathy, other

Rheumatological: Fibromyalgia, osteoarthritis, rheumatoid arthritis, whiplash, sarcoidosis, connective tissue disease (Marfan etc.), rheumatoid polymyalgia, other

b= Percentages of psychiatric illnesses are of total psychiatric patients and not total patients. c=Midline percentage of open surgery or converted to open. d=Other pathology being bleeding, non-traumatic spleen rupture, anastomotic leakage, intraabdominal abscess, or laparotomy with no pathology found. e = The mortality within 180 days postoperatively

A comparison of respondents and non-respondents at each postoperative timepoint is presented in Supplementary Table 1. Overall, respondents and non-respondents were largely comparable across baseline characteristics, including sex, ASA physical status, and CFS, although respondents at POD 30 were younger than non-respondents (median 69.8 vs. 75.5 years, *p* = 0.035). Non-respondents at POD 90 and POD 180 had significantly higher mortality rates compared with respondents.

### Degree and subjects of worry

Overall, 36.1% of patients reported a hDOW at discharge, 22.4% on POD 30, 45.0% on POD 90, and 32.0% on POD 180. The median DOW (IQRs) and the subjects of worry at all timepoints are presented in Table 2.

**Table 2:** Degree and subjects of worry at discharge, 30, 90, and 180 days postoperatively

Number of patients (*N*=)	At discharge	POD30	POD90	POD180
	61	156	60	25
Median (interquartile range) DOW	5 (3–7)	3.5 (2–5)	5 (1–7)	5 (2–6)
High DOW^a^	22 (36.1)	35 (22.4)	27 (45.0)	8 (32.0)
Total^b^ (N=)	107	183	72	37
Functional level	18 (16.8)	15 (8.2)	7 (9.7)	5 (13.5)
Mental status	2 (1.9)	9 (4.9)	3 (4.2)	2 (5.4)
Pain	14 (13.1)	5 (2.7)	1 (1.4)	1 (2.7)
Relapse	9 (8.4)	42 (23.0)	15 (20.8)	7 (18.9)
Disease	10 (9.3)	25 (13.7)	10 (13.9)	8 (21.6)
Death	2 (1.9)	2 (1.1)	2 (2.8)	0 (0.0)
Procedure/treatment	3 (2.8)	15 (8.2)	9 (12.5)	2 (5.4)
Daily living^c^	10 (9.3)	10 (5.5)	6 (8.3)	8 (21.6)
Stoma	4 (3.7)	9 (4.9)	7 (9.7)	0 (0.0)
Future	11 (10.3)	18 (9.8)	2 (2.8)	1 (2.7)
Food intake^d^	6 (5.6)	6 (3.3)	2 (2.8)	3 (8.1)
Test results^e^	2 (1.9)	5 (2.7)	2 (2.8)	0 (0.0)
Postoperative symptoms and complications	5 (4.8)	18 (9.8)	4 (5.6)	0 (0.0)
Unknowingness during/after hospitalization and rehabilitation about postoperative course^f^	11 (10.3)	4 (2.2)	2 (2.8)	0 (0.0)

Values are number of patients (%) unless stated otherwise. POD = postoperative day. DOW = Degree of Worry. DOW-scale shows patients’ level of worry between 0 and 10, with 0 being the minimal level of worry

**a =** DOW 6–10

**b=** Total number of reported subjects of worry (patients could report up to three subjects of worry each)

**c= Daily living**: Refers to the psychological burden of managing everyday functioning under uncertainty

**d= Food intake**: Refers to patient worry regarding postoperative eating, including uncertainty about appropriate food choices, fear of digestive discomfort, and challenges related to nutritional intake following surgery

**e= Test results**: Encompasses anxiety related to postoperative paraclinical testing, such as imaging (CT, PET-CT) and blood analyses, particularly regarding the interpretation, timing, and implications of results

**f= Unknowingness during/after hospitalization and rehabilitation about postoperative course**: Captures patients’ experiences of uncertainty during hospitalization and rehabilitation, specifically related to insufficient information about the expected postoperative and recovery trajectory

**Fig. 2:**
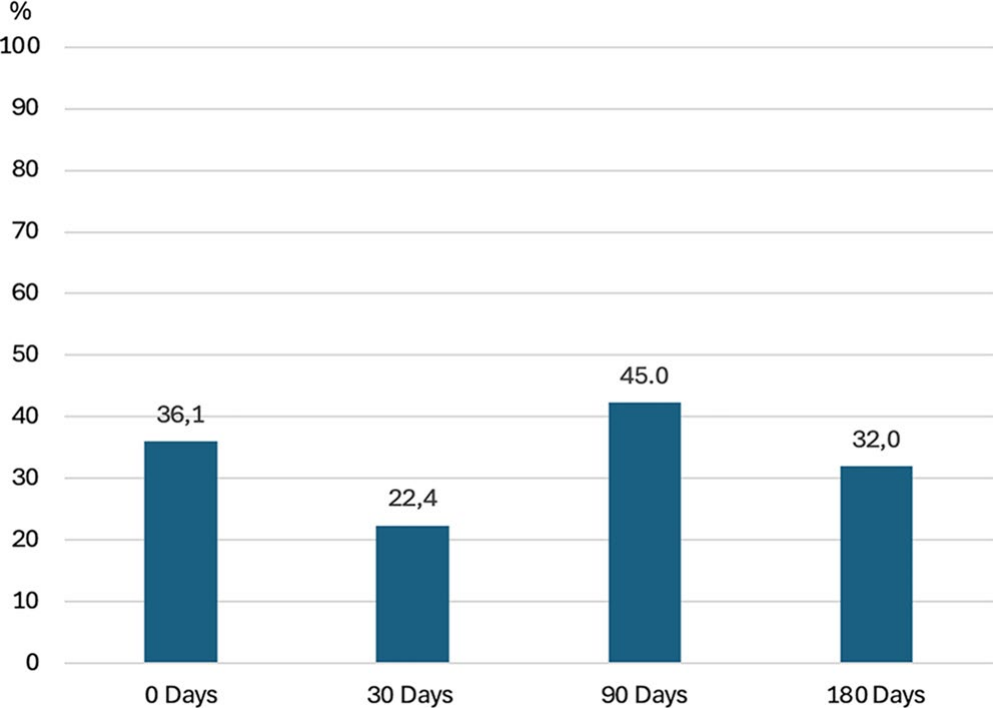
Percentage of patients with hDOW (Degree of worry ≥ 6) across different time points (Discharge, POD 30, 90, and 180). The Y-axis represents the percentage of patients, while the X-axis indicates the days of follow-up. The bars illustrate the variation in the proportion of patients achieving a high DOW over time providing insight into the distribution of hDOW across postoperative timepoints

Figure 2 represents proportion of patient with hDOW at different timepoints.

**Fig. 3:**
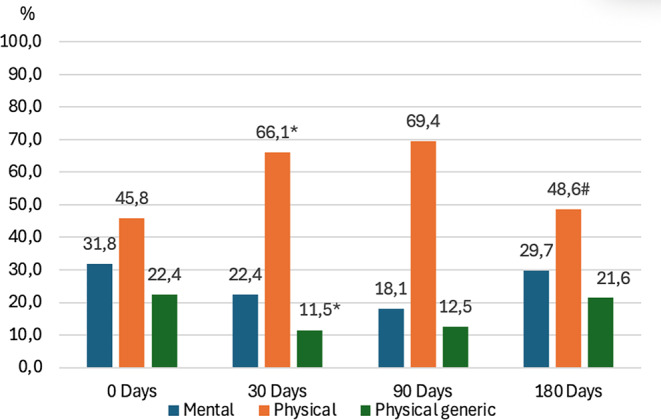
Percentages of subjects of worry across the domains *Physical*, *Mental*, and *Physical-Generic* at discharge, POD 30, 90, and 180. Significant differences (*P* < 0.05) between days 0 and 30 are denoted by an asterisk (*), while significant differences (*P* < 0.05) between days 30 and 180 are indicated by a hash symbol (#). The data highlight differences in domain-specific distribution across timepoints

Significant variation across postoperative timepoints was observed in the distribution of worry domains (Fig. 3).

The proportion of physical worry was higher at POD 30 than at discharge and lower at POD 180. Physical-generic worry showed a similar distribution across timepoints, whereas the proportion of mental worry varied without a statistically significant pattern.

### Days alive and out of hospital and readmission

Cumulative DAOH increased progressively with longer follow-up. Median cumulative DAOH was 21 days (IQR 4–26) at day 30, increasing to 80 days (IQR 59.5–86) at day 90 and 170 days (IQR 142–176) at day 180, reflecting greater time spent alive and out of hospital at later postoperative timepoints.

When recovery was examined within specific postoperative intervals, median incremental DAOH was 21 days (IQR 4–26) from discharge to POD 30 (DAOH0–30), 60 days (IQR 59–60) between POD 30 and POD 90 (DAOH30–90), and 90 days (IQR 90–90) between POD 90 and POD 180 (DAOH90–180).

Using predefined thresholds to identify low recovery, the proportion of patients with reduced DAOH declined over time, from 37.1% at POD 30 to 24.9% at POD 90 and 20.4% at POD 180.

Friedman’s test demonstrated significant differences in DAOH across the three incremental intervals (*p* < 0.001), with all pairwise comparisons reaching statistical significance (*p* < 0.001).

No significant association was found between hDOW at discharge and DAOH0-30 (hDOW: 19.0 days (3.0–25.0) vs. non-hDOW: 22.5 days (0–27.0), *p* = 0.51). Similarly, hDOW at POD 90 was not associated with DAOH90-180 (hDOW: 90.0 days (90.0–90.0) vs. non-hDOW: 90.0 days (90.0–90.0), *p* = 0.65) nor with cumulative DAOH180 (hDOW: 170.0 days (149.0–177.0) vs. non-hDOW: 174.0 days (155.0–177.0), *p* = 0.46). In contrast, hDOW at POD 30 showed a tendency toward reduced DAOH30-90 (hDOW: 60.0 days (22.0–60.0) vs. non-hDOW: 60.0 days (60.0–60.0), *p* = 0.06). Although median DAOH30-90 was identical between groups, the interquartile ranges differed substantially, suggesting greater variability in recovery among hDOW patients. A statistically significant difference was observed in cumulative DAOH by POD 90 between patients with and without hDOW at POD 30, with hDOW patients spending fewer DAOH (hDOW: 73.0 days (22.0–85.0) vs. non-hDOW: 83.0 days (68.0–86.0), *p* = 0.008).

No significant differences were observed between hDOW at POD 180 and DAOH90-180 (hDOW: 90 days (62.5–90.0) vs. non-hDOW: 90 days (90.0–90.0), *p* = 0.56) or cumulative DAOH180 (hDOW: 165.0 days (150.3–173.8) vs. non-hDOW: 172.0 days (131.5–177.0), *p* = 0.48).

Finally, no significant associations were detected between hDOW at discharge and readmission within 30 days (*p* = 0.889), between hDOW at POD 30 and readmission within 90 days (*p* = 0.250), or between hDOW at POD 30 and readmission within 180 days, although the latter showed a non-significant trend (*p* = 0.057).

## Discussion

We present an analysis of the degree and subjects of worry during the 180-day postoperative period following major abdominal emergency surgery. The study found that up to one in three patients remained to have a hDOW up to 180 days postoperatively, underscoring that hDOW persists well beyond hospital discharge in this high-risk population. In addition, the proportion of physical worries was significantly higher at POD 30 (66.1%) than at discharge (22.4%) and significantly lower at POD 180, highlighting that the distribution of patient-reported worry differed across postoperative timepoints.

The DOW questionnaire was deliberately chosen over more established and validated instruments such as the EQ-5D [24] and SF-36 [25]. While these instruments provide comprehensive assessments of health-related quality of life, they do not capture the specific subjects of worry, which was a primary focus of this study. The DOW questionnaire is specifically designed to quantify patients’ subjective perception of worry and urgency and uniquely enables identification and categorization of the specific subjects of worry expressed by patients. Furthermore, its brevity made it particularly suitable for the telephone-based follow-up format used in this study, minimizing patient burden. We therefore argue that DOW represents the most appropriate instrument for investigating patient-reported worry as a distinct and clinically relevant phenomenon in the postoperative setting.

A key finding of this study was the association between hDOW assessed at POD 30 and reduced cumulative DAOH by POD 90. Patients reporting hDOW at POD 30 spent significantly fewer DAOH during the subsequent recovery period compared with patients reporting low DOW (*p* = 0.008). This finding suggests that patient-reported DOW at POD 30 represents a clinically relevant marker of impaired postoperative recovery. Notably, no corresponding associations were observed for hDOW assessed at discharge or at later postoperative timepoints, emphasizing that the timing of worry assessment appears critical, with POD 30 constituting a particularly meaningful window in the recovery. The incremental DAOH analysis demonstrated a non-significant trend toward reduced recovery between POD 30 and POD 90 among patients with hDOW at POD 30 (*p* = 0.06). Although this finding did not reach statistical significance, it supports the primary cumulative DAOH result and suggests that hDOW in the early recovery phase may be associated with a slower recovery rate. This observation should be interpreted as a secondary, hypothesis-generating finding that warrants further investigation in larger cohorts with paired longitudinal data. It should be noted that POD 30 also had the highest response rate (156/333, 46.8%) compared with discharge (61/342, 17.8%), POD 90 (60/324, 18.5%), and POD 180 (25/313, 8.0%). The statistical power to detect an association between hDOW and DAOH was therefore greater at POD 30 than at other timepoints, and the absence of significant associations at other timepoints may partly reflect limited statistical power rather than a true absence of association. The finding at POD 30 should therefore be interpreted with caution.

No significant associations were identified between hDOW at discharge and subsequent DAOH, nor between hDOW assessed at POD 90 or POD 180. The lack of association at discharge may reflect the immediate postoperative context, where DOW is influenced by acute surgical stress, pain, and uncertainty, but does not necessarily translate into longer-term functional impairment. Similarly, the absence of associations at later timepoints may reflect ceiling effects in DAOH, psychological adaptation over time, or reduced variability in recovery among surviving patients.

Beyond overall DOW, the subjects of worry varied across postoperative timepoints. The proportion of physical worry was higher at POD 30 than at discharge and lower at POD 180, whereas the proportion of mental worry varied across timepoints without a statistically significant pattern. This difference in the distribution of worry across timepoints aligns with prior work indicating that psychological well-being may improve as physical symptoms decrease and autonomy is regained during postoperative recovery [26]. Existing literature also shows that emergency surgery is associated with higher anxiety due to the sudden and unplanned nature of the event, with some studies reporting improvement postoperatively [27]. Furthermore, patients with prolonged recoveries after emergency surgery may experience more severe psychological consequences, including persistent anxiety-related symptoms [28]. A recent study also suggests that most patients return to normal *health-related quality of life* (HRQoL) within 180 days following emergency laparotomy, supporting the interpretation that distress and worry may change across recovery phases [29].

Previous research has established that patient-reported DOW is associated with acute care processes and outcomes and may reflect perceived urgency and illness severity [11–13]. In emergency care settings, DOW has been applied in triage contexts and has shown potential in supporting clinical assessment and decision-making [10–15]. Our findings extend this literature by suggesting that patient-reported DOW during postoperative recovery, particularly at POD 30, may also be relevant for identifying patients at risk of poorer functional recovery, as reflected by reduced DAOH by POD 90.

The observed association between hDOW at POD 30 and reduced cumulative DAOH by POD 90 is consistent with prior studies demonstrating that DAOH increases over time across diverse surgical populations, including patients undergoing hip fracture repair, emergency laparotomy, cardiac surgery, and heart transplantation [30–33]. Moreover, low DAOH in the early postoperative period has been associated with adverse longer-term outcomes in several settings, underscoring the prognostic relevance of early postoperative recovery [30–33]. In this context, DOW assessed at POD 30 may capture patient-perceived vulnerability that is not fully reflected by traditional clinical measures.

This study’s strengths include its prospective cohort design, ensuring high-quality and longitudinal data over six months. The 0–10-point DOW scale enabled nuanced measurement of worry, while categorizing subjects of worry into physical, mental, and physical-generic domains facilitated clinically meaningful interpretation of the subjects of patients worry. However, this study has several limitations. First, this was a single-center study conducted at a tertiary care hospital, which may limit the generalizability of the findings to other hospitals and healthcare systems. Second, no preoperative baseline DOW was measured, which limits the ability to assess changes in DOW following surgery. Given that the study population was older with a median CFS of 4 (“vulnerable”), it is possible that baseline DOW was already elevated prior to surgery. However, CFS measures functional status rather than subjective worry, and the relationship between preoperative functional status and DOW is not established. In particular, preoperative concerns related to physical and physical-generic domains, such as functional level, pain, and food intake, may already have been present prior to surgery, potentially influencing the observed distribution of worry domains in the postoperative period. Future studies should consider incorporating preoperative DOW assessments to enable comparison with postoperative levels.

Third, response rates declined substantially at later follow-up timepoints, particularly at POD 90 and 180, which may introduce selection bias. A formal comparison of respondents and non-respondents at each timepoint revealed that non-respondents at POD 90 and POD 180 had significantly higher mortality rates, suggesting a survivor bias that may have influenced the observed worry patterns at later timepoints. Respondents and non-respondents were otherwise comparable on baseline characteristics, including age, sex, ASA, and CFS (Supplementary Table 1).

In addition, only a subset of patients completed the DOW assessment at discharge, resulting in smaller sample sizes at this timepoint. Furthermore, the response rate varied substantially across timepoints, with POD 30 having the highest response rate (156/333, 46.8%) compared with discharge (61/342, 17.8%), POD 90 (60/324, 18.5%), and POD 180 (25/313, 8.0%). The greater statistical power at POD 30 may have contributed to the detection of a significant association with DAOH, and the absence of associations at other timepoints should not be interpreted as evidence of no effect. Fourth, DOW responses were not paired across follow-up assessments, precluding within-subject repeated-measures analyses and limiting the ability to describe individual patterns of worry over time. Analyses therefore relied on independent cross-sectional samples at each timepoint. Fifth, reliance on self-reported measures introduces potential sources of bias, including recall bias and social desirability bias [34]. Sixth, the absence of socioeconomic data restricted the ability to examine the influence of social and economic factors on patient-reported worry and postoperative outcomes. Seventh, the expansion of the DOW scale from 1 to 10 to 0–10 and the definition of hDOW as ≥ 6 were established in collaboration with the DOW developers but have not been formally validated in a postoperative population. The addition of 0 was intended to allow patients to report absence of worry and to reduce the risk of a floor effect in a follow-up setting where no current worry is plausible. However, this modification limits direct comparability with previous DOW studies using the original 1–10 scale, and the hDOW threshold should therefore be regarded as exploratory and hypothesis-generating. Finally, the categorization of patient-reported subjects of worry was exploratory and not based on a validated classification system. Coding was performed by a single investigator, with uncertain cases discussed with a senior investigator to ensure agreement on domain placement. Although domain assignments were agreed upon by all authors through clinical consensus, formal inter-rater reliability was not assessed. This may limit the reproducibility and comparability of the worry-domain framework with other studies.

Despite these limitations, the study provides valuable insights into patterns of patient-reported worry following major emergency abdominal surgery and highlights the complexity of worry as a patient-reported outcome with potential relevance for postoperative follow-up and recovery monitoring.

## Conclusion

The DOW following major emergency abdominal surgery differed across postoperative timepoints. Although the overall proportion of patients reporting hDOW remained relatively stable over 180 days, with approximately one in three patients experiencing hDOW, the subjects of worry changed across postoperative timepoints. The proportion of physical worry was higher at POD 30 than at discharge and lower at POD 180, while the proportion of mental worry varied across timepoints. In parallel, DAOH increased over time, reflecting postoperative recovery. Notably, hDOW at POD 30 was associated with fewer DAOH by POD 90. Together, these findings highlight the importance of recognizing patient-reported DOW as a clinically relevant aspect of postoperative recovery that differed across timepoints. However, given the observational design, independent cross-sectional samples, exploratory worry-domain coding, and modest sample sizes at several timepoints, these findings should be regarded as hypothesis-generating and warrant confirmation in larger, prospective studies with paired longitudinal data. 

## Supplementary Information

Below is the link to the electronic supplementary material.

Supplementary Material 1

## Data Availability

The dataset generated and analyzed in the study is not publicly available due to Danish protection guidelines. Information about the data and analyses can be obtained upon request from the corresponding author.
