# Supplementary material for: Patterns of patient worry following major emergency abdominal surgery: a 180-day follow-up study
Source: Eur J Trauma Emerg Surg. 2026 Jul 29;52(1):234. doi: 10.1007/s00068-026-03287-1 (PMC13421358; doi:10.1007/s00068-026-03287-1)
Supplement: Supplementary file 1 — Supplementary Material 1 [file 68_2026_3287_MOESM1_ESM.docx]

**Supplementary Table 1. Comparison of respondents and non-respondents at each postoperative timepoint**

| **Variable** | **Discharge n = 61 vs. 317** | | **POD 30 n = 156 vs. 222** | | **POD 90 n = 60 vs. 318** | | **POD 180 n = 25 vs. 353** | |
| --- | --- | --- | --- | --- | --- | --- | --- | --- |
|  | *R* | *NR / p* | *R* | *NR / p* | *R* | *NR / p* | *R* | *NR / p* |
| **Baseline characteristics** |  |  |  |  |  |  |  |  |
| Age, median (IQR), years | 71.9 (49.8-78.8) | 73.0 (59.7-81.9) / p= 0.156 | 69.8 (54.2-79.3) | 75.5 (59.6-81.3) / 0.035 | 70.9 (54.1-79.3) | 73.5 (58.2-81.1) / 0.288 | 70.8 (51.4-78.8) | 73.3 (58.5-81.0) / 0.379 |
| Sex, male, (n=%) | 25 (41.0) | 140 (44.2) / p= 0.65 | 60 (42.6) | 105 (44.3) / 0.740 | 22 (36.7) | 143 (45.0) / 0.243 | 11 (44.0) | 154 (43.6) / 0.971 |
| **ASA physical status** |  |  |  |  |  |  |  |  |
| I–II, (n=%) | 40 (65.6) | 170 (53.6) | 83 (58.9) | 127 (53.6) | 38 (63.3) | 172 (54.1) | 15 (60.0) | 195 (55.2) |
| III, (n=%) | 18 (29.5) | 128 (40.4) | 54 (38.3) | 92 (38.8) | 22 (36.7) | 124 (39.0) | 10 (40.0) | 136 (38.5) |
| IV–V, (n=%) | 3 (4.9) | 19 (6.0) / p= 0.508 | 4 (2.8) | 18 (7.6) / p= 0.363 | 0 (0.0) | 22 (6.9)/ p= 0.264 | 0 (0.0) | 22 (6.2) / p= 0.725 |
| **Clinical Frailty Scale** |  |  |  |  |  |  |  |  |
| 1–3, (n=%) | 42 (68.9) | 185 (58.5) | 95 (67.9) | 132 (56.4) | 45 (76.3) | 182 (57.4) | 19 (76.0) | 208 (59.3) |
| 4–6, (n=%) | 18 (29.5) | 126 (39.9) | 44 (31.4) | 100 (42.4) | 14 (23.7) | 130 (41.0) | 6 (24.0) | 138 (39.3) |
| 7–9, (n=%) | 0 (0.0) | 5 (1.6) / p= 0.362) | 1 (0.7) | 4 (1.7) / p= 0.095 | 0 (0.0) | 5 (1.6) / p= 0.083 | 0 (0.0) | 5 (1.4) / p= 0.333 |
| **Outcomes** |  |  |  |  |  |  |  |  |
| DAOH, median (IQR) |  |  | 22 (4.0-26.0) | 20 (3.3-26.0) / p= 0.345 | 83 (66.5-87.0) | 80 (57.5-86.0) / p= 0.087 | 166 (151.5-176) | 170 (141.0-176.0) / p= 0.967 |
| Mortality, (n=%) |  |  | 5 (8.2) | 43 (13.6) / p= 0.249 | 3 (2.0) | 53 (22.4) / p= <0.001 | 1 (1.7) | 64 (20.1) / p= <0.001 |

Values are number of patients (%) unless stated otherwise. R = respondents; NR = non-respondents. Continuous variables compared using Mann-Whitney U test and reported as median (interquartile range). Categorical variables compared using Pearson chi-squared test. p-values < 0.05 considered statistically significant. ASA = American Society of Anesthesiologists. CFS = Clinical Frailty Scale. DAOH = Days Alive and Out of Hospital.

Cumulative DAOH for the interval corresponding to each assessment timepoint: DAOH0–30 at POD 30, DAOH0–90 at POD 90, and DAOH0–180 at POD 180.

Mortality reflects the proportion of patients who died within 30, 90, and 180 days after hospital discharge, compared between respondents and non-respondents at the preceding timepoint
